# Quinia Pills

**Published:** 1874-11

**Authors:** 


Pills of Sulphate of Quinia.—The following is the formula:

| | |
|---|---|
| Take of Sulphate of Quinia, . . . | 600 grs. |
| Tartaric Acid, . . . . . | 100 grs. |
| Glycerine, pure, . . . . . | 75 minims. |

Rub the quinia and acid together in a mortar to a fine powder, till no appearance of crystals remains, add the glycerin—just 75 minims, no more nor less—and continue the trituration till the powder becomes adherent, when it should be beaten into proper form for handling and divided into the required number of pills. The mass is firm, solid, rolls well, does not set for some hours; is, in fact, a "beautiful mass," and the pills will be found quite small for their weight, very white, if rolled in starch powder, and however old or dry they may become, they remain perfectly and entirely soluble.—*American Journal of Pharmacy.*

